# Supplementary figures and images for: Calcium signaling through a transient receptor channel is important for Toxoplasma gondii growth
Source: eLife. 2021 Jun 9;10:e63417. doi: 10.7554/eLife.63417 (PMC8216714; doi:10.7554/eLife.63417)

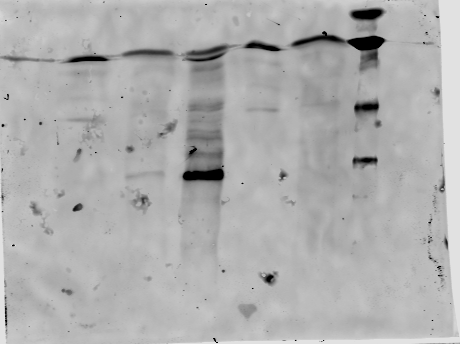

Supplement: Source data 1. [file elife-63417-data1.zip › Source data 1. Original Blots/Raw data/Figure 1 Source Data 1 Panel C Raw.tif]

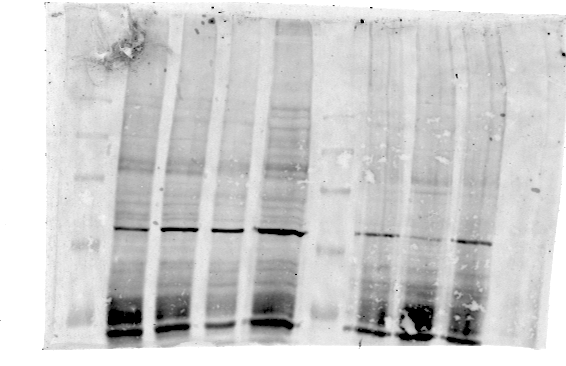

Supplement: Source data 1. [file elife-63417-data1.zip › Source data 1. Original Blots/Raw data/Figure 2 Supplementary 1 Source Data 1 Panel B Raw a.tif]

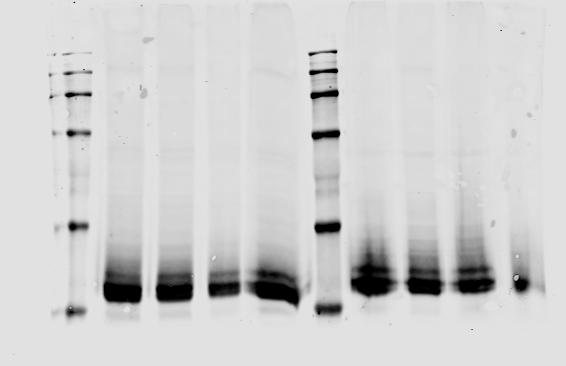

Supplement: Source data 1. [file elife-63417-data1.zip › Source data 1. Original Blots/Raw data/Figure 2 Supplementary 1 Source Data 2 Panel B Raw b.tif]

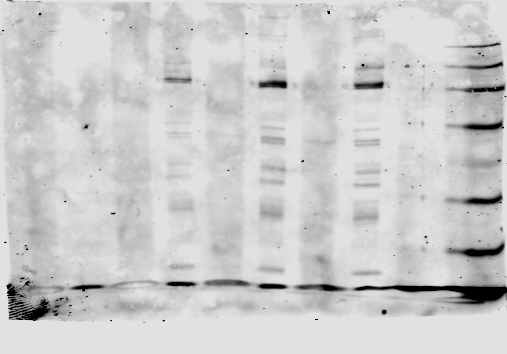

Supplement: Source data 1. [file elife-63417-data1.zip › Source data 1. Original Blots/Raw data/Figure 1 Supplementary 1 Source Data 1 Panel C Raw.tif]

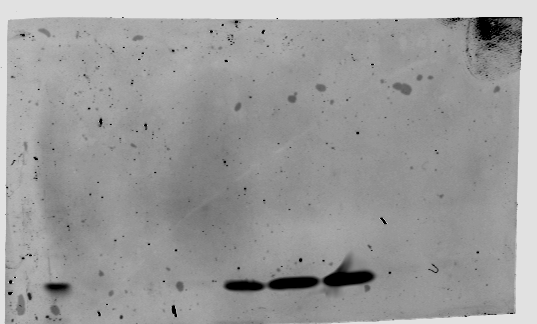

Supplement: Source data 1. [file elife-63417-data1.zip › Source data 1. Original Blots/Raw data/Figure 2 Supplementary 1 Source Data 3 Panel C Raw c.png]

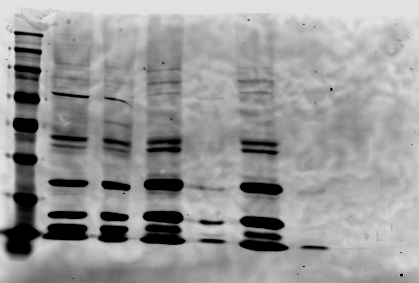

Supplement: Source data 1. [file elife-63417-data1.zip › Source data 1. Original Blots/Raw data/Figure 2 Supplementary 1 Source Data 1 Panel C Raw a.tif]

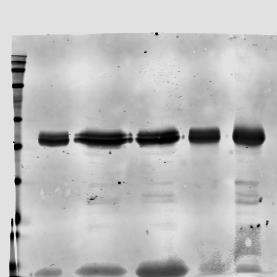

Supplement: Source data 1. [file elife-63417-data1.zip › Source data 1. Original Blots/Raw data/Figure 2 Supplementary 1 Source Data 2 Panel C Raw b.png]

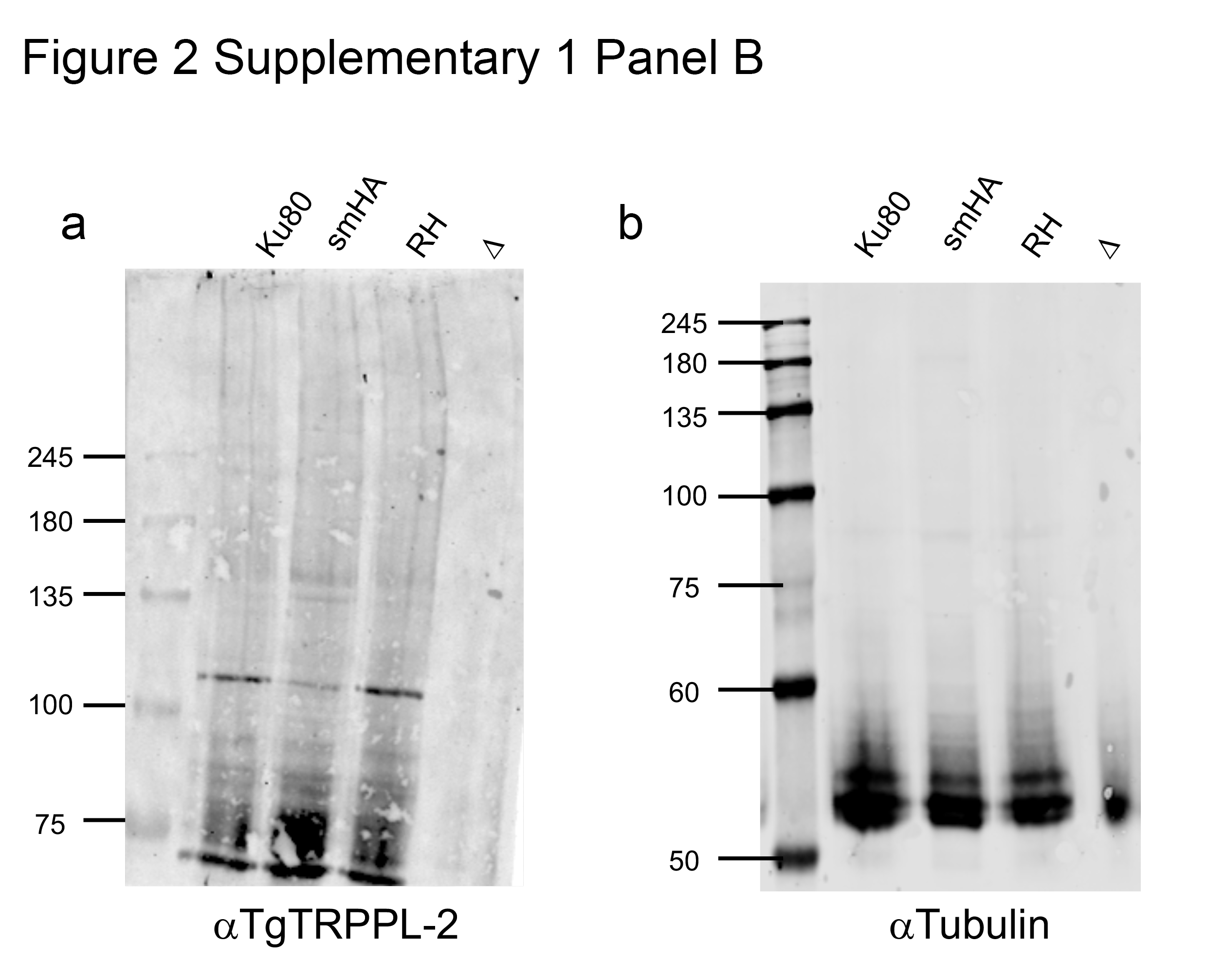

Supplement: Source data 1. [file elife-63417-data1.zip › Source data 1. Original Blots/TIFF/Figure 2 Supplementary 1 Source Data 1 Panel B (Karla M. Ma╠ürquez Nogueras's conflicted copy 2021-06-02).tif]

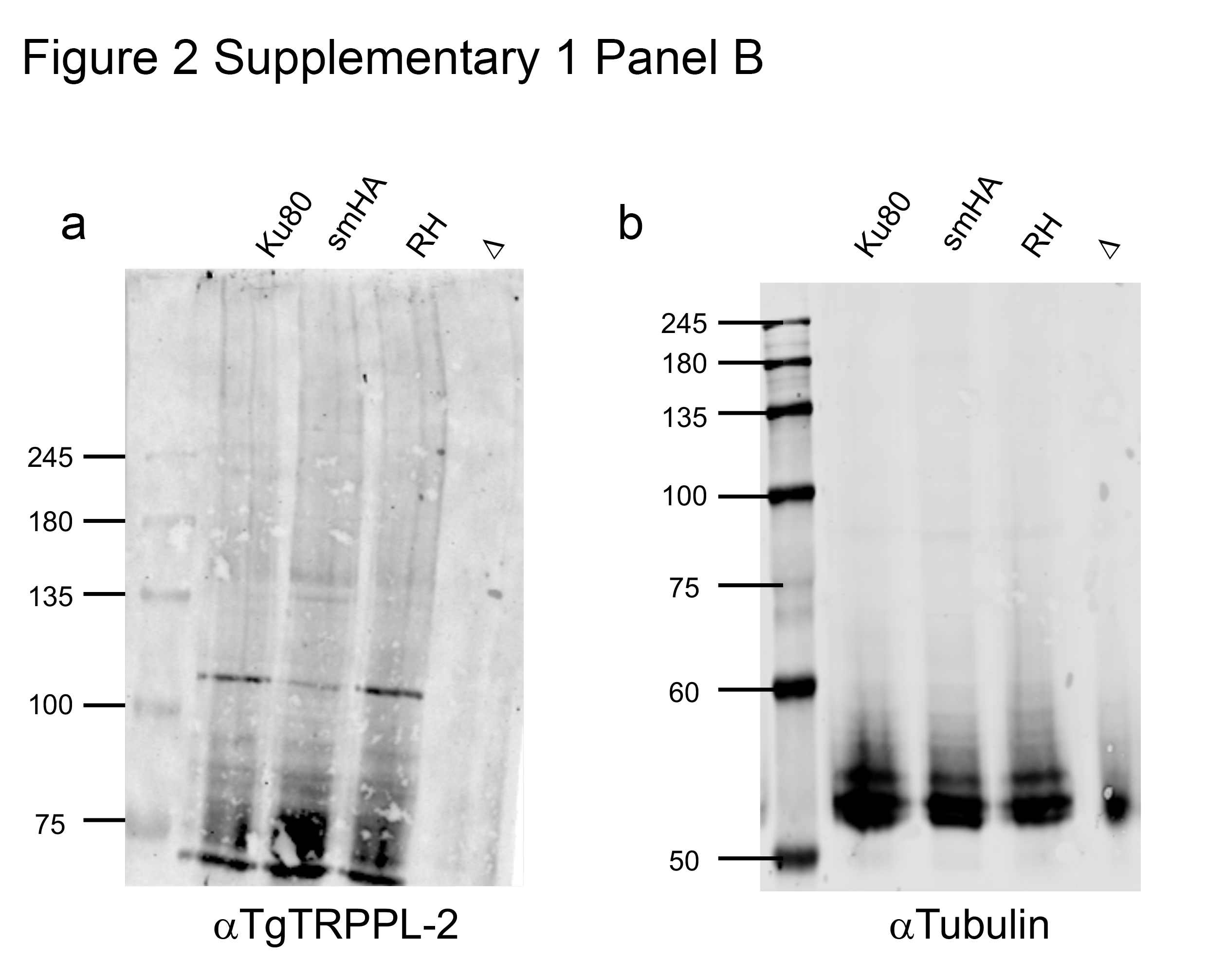

Supplement: Source data 1. [file elife-63417-data1.zip › Source data 1. Original Blots/TIFF/Figure 2 Supplementary 1 Source Data 1 Panel B.tif]

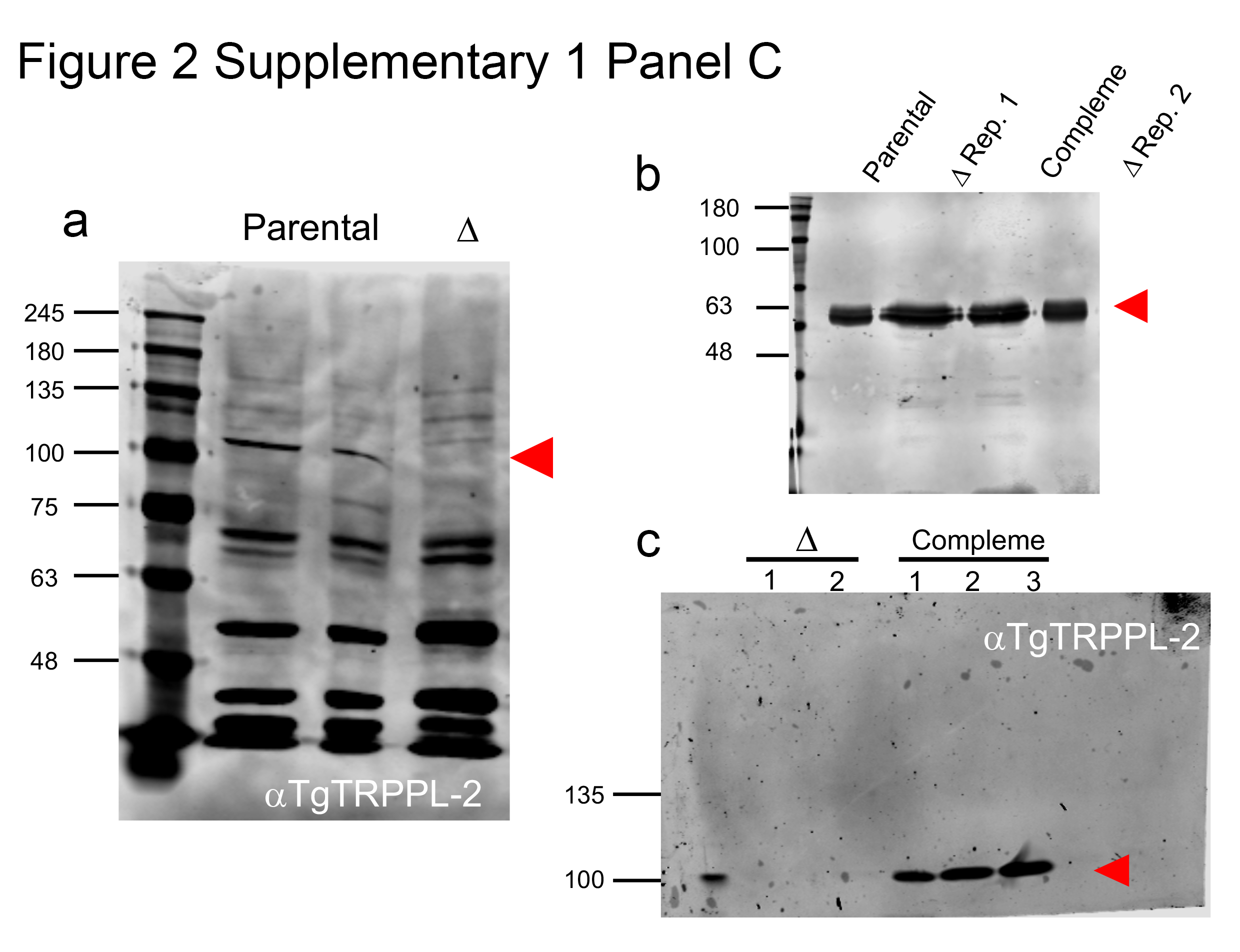

Supplement: Source data 1. [file elife-63417-data1.zip › Source data 1. Original Blots/TIFF/Figure 2 Supplementary 1 Source Data 2 Panel C.tif]

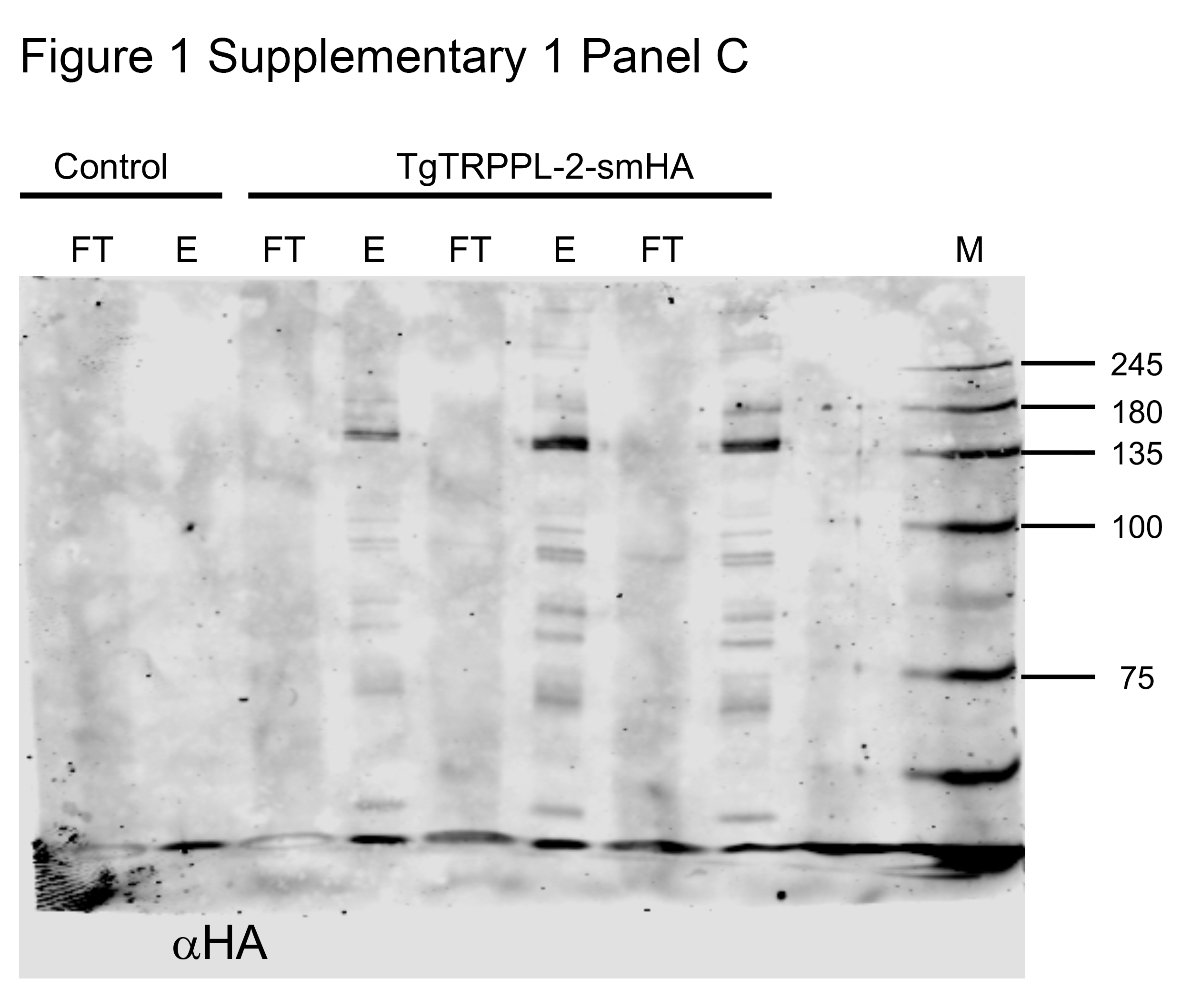

Supplement: Source data 1. [file elife-63417-data1.zip › Source data 1. Original Blots/TIFF/Figure 1 Supplementary 1 Source Data 1 Panel C.tif]

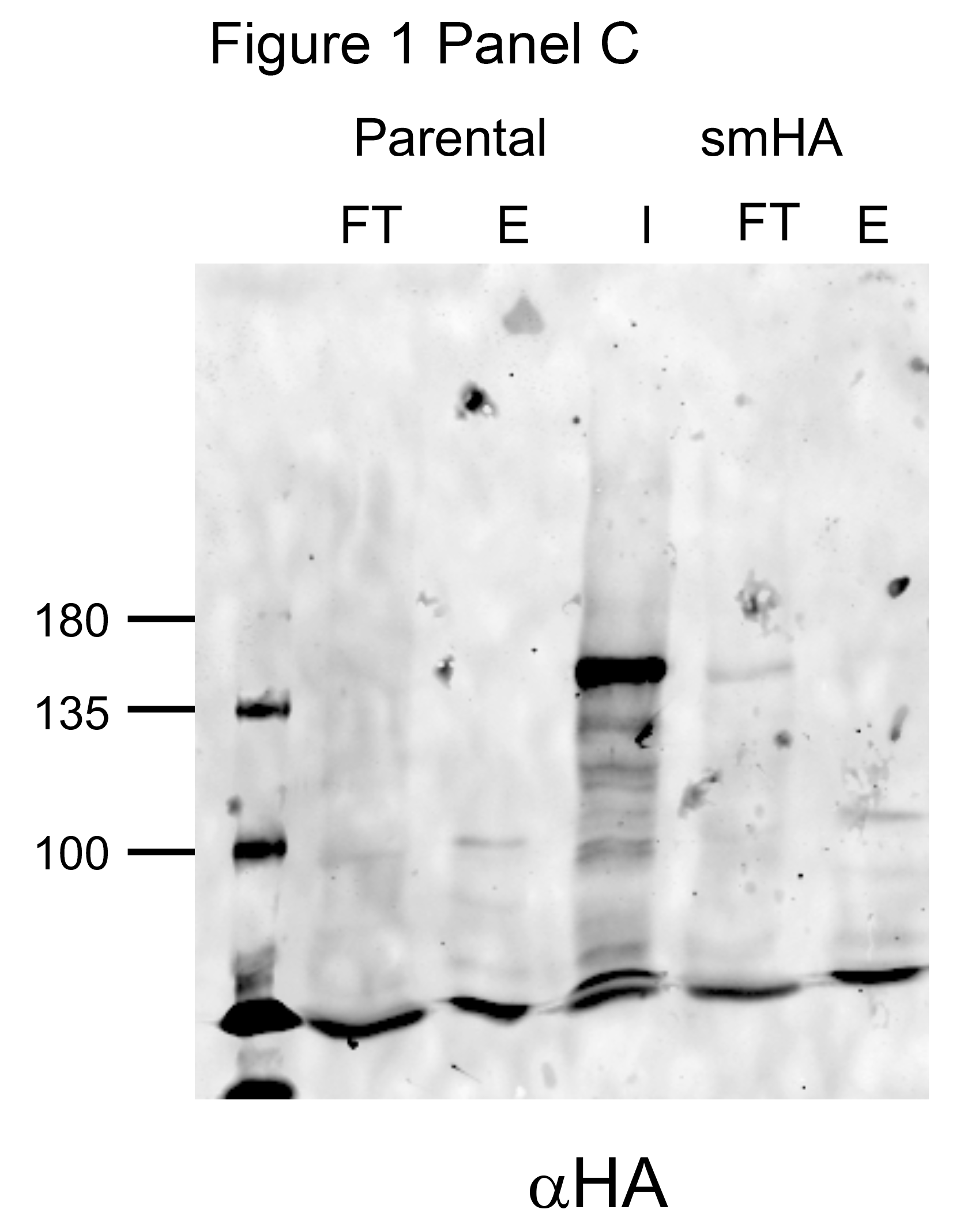

Supplement: Source data 1. [file elife-63417-data1.zip › Source data 1. Original Blots/TIFF/Figure 1 Source Data 1 Panel C.tif]
