# Supplementary material for: Calcium signaling through a transient receptor channel is important for Toxoplasma gondii growth
Source: eLife. 2021 Jun 9;10:e63417. doi: 10.7554/eLife.63417 (PMC8216714; doi:10.7554/eLife.63417)
Supplement: Supplementary file 1. [file elife-63417-supp1.docx]

**Calcium signaling by a Transient Receptor Channel is important for *Toxoplasma gondii* growth**

**Márquez-Nogueras et al**

**Supplementary File 1:** Top 10 hits of HHPRED analysis of TgTRPPL-2.

| **Name** | **Probability** | **E-value** | **AA length** | **Organism** | **PDB Reference** |
| --- | --- | --- | --- | --- | --- |
| **Polycystic kidney disease 2-like 1** | 99.45 | 7.10 E^-11^ | 805 | *H. sapiens* | 6DU8_A |
| **Polycystin-2; PKD2** | 99.44 | 1.10 E^-10^ | 756 | *H. sapiens* | 6WB8_D |
| **Polycystic kidney disease 2-like 1** | 99.39 | 2.10 E^-10^ | 566 | *M. musculus* | 5Z1W_C |
| **Polycystin-2, Polycystin-1** | 99.38 | 2.40 E^-10^ | 577 | *H. sapiens* | 6A70_A |
| **Polycystin-2** | 99.35 | 2.40 E^-10^ | 968 | *H. sapiens* | 5MKE_A |
| **Polycystin-2, Polycystin-1** | 99.3 | 2.80 E^-10^ | 1153 | *H. sapiens* | 6A70_B |
| **Polycystin-2; TRP channel, PKD2** | 99.22 | 3.30 E^-09^ | 510 | *H. sapiens* | 5T4D_A |
| **TRPV2; Transport protein, TRP channel** | 98.52 | 6.6 E^-05^ | 613 | *O. cuniculus* | 5AN8_B |
| **Mucolipin-3; TRP channel, lysosomal** | 98.31 | 0.00031 | 558 | *H. sapiens* | 6AYF_C |
| **Transient receptor potential cation channel** | 98.28 | 0.00015 | 639 | *M. musculus* | 6LGP_D |
