# Supplementary material for: Calcium signaling through a transient receptor channel is important for Toxoplasma gondii growth
Source: eLife. 2021 Jun 9;10:e63417. doi: 10.7554/eLife.63417 (PMC8216714; doi:10.7554/eLife.63417)
Supplement: Supplementary file 2. [file elife-63417-supp2.docx]

**Calcium signaling by a Transient Receptor Channel is important for *Toxoplasma gondii* growth**

**Márquez-Nogueras et al**

**Supplementary File 2:** List of Mass spectrometry hits by TgTRPPL-2 Immunoprecipitation*.

| **Gene ID** | **Description** | **Phenotype^#^** | **TMD** | **Average peptide counts^&^** |
| --- | --- | --- | --- | --- |
| TgGT1_310560 | Hypothetical protein (TgTRPPL-2) | -2.49 | 13 | 3 |
| TgGT1_247370 | Hypothetical protein (TgTRPPL-1) | -1.42 | 13 | 3 |
| TgGT1_214300 | Hypothetical Protein | -0.36 | 9 | 3 |
| TgGT1_280560 | Selenide, water dikinase | 0.07 | 0 | 6 |
| TgGT1_201680 | Putative eukaryotic initiation factor-3 subunit 10 | -4.55 | 0 | 6 |
| TgGT1_228170 | Inner membrane complex protein IMC2A | -3.28 | 1 | 5.5 |
| TgGT1_212300 | Hypothetical protein | 0.69 | 1 | 3 |
| TgGT1_229180 | HEAT repeat-containing protein | -5.05 | 0 | 3 |

*See Material and Methods for the protocol.

**^#^** Fitness score for each gene was obtained from ToxoDB.

**^&^** Number of peptides identified are shown
