# Supplementary material for: Calcium signaling through a transient receptor channel is important for Toxoplasma gondii growth
Source: eLife. 2021 Jun 9;10:e63417. doi: 10.7554/eLife.63417 (PMC8216714; doi:10.7554/eLife.63417)
Supplement: Supplementary file 3. [file elife-63417-supp3.docx]

**Calcium signaling by a Transient Receptor Channel is important for *Toxoplasma gondii* growth**

**Márquez-Nogueras et al**

**Supplementary File 3:** Primers used in this work

| ***Endogenous Tagging of TgTRPPL2*** | | |
| --- | --- | --- |
| T1 | TgTRPPL2_pLic_F | TACTTCCAATCCAATTTAATGCGAGAAGCGCATT GAGGAATGG |
| T2 | TgTRPPL2_pLic_F | TCCTCCACTTCCAATTTTAGCCTCTTCTCCCAGG ATGTTGACGC |
| T3 | TgTRPPL2_Validation_Tag_F | TATGTGTGCCTGCCTGCGCAT |
| ***Disruption of TgTRPPL2*** | | |
| K1 | TgTRPPL2_Cas9_gRNA_F | TATGTCACATGTCTTTTCTCGTTTTAGAGCTAGAA  ATAGCAAG |
| K2 | TgTRPPL2_DHFR_F | CTTTGGTTTCCCTCTCTCGTCCATGAAGCTTCGCC AGGCTGTAAATCC |
| K3 | TgTRPPL2_DHFR_R | TGGACGCCCAGCTCGACATGTCATCCTGCAAGTG CATAGAAGGA |
| K4 | TgTRPPL2_Validation R | CGATGAGGTGGATGTAGCTGAATG |
| ***RT-PCR of TgTRPPL2*** | | |
| Q1 | TgTRPPL2_qPCR_F | GAGCTCCGACGCAGGCCAGCAG |
| Q2 | TgTRPPL2_qPCR_R | CCCGGGCGATGAGGTGGATGTAGCTGAATG |
| ***Cloning for Heterologous expression in HEK293-3KO cells*** | | |
| C1 | pCDNA3_TgTRPPL2_F | cagatatccatcacactggcATGCATGCATTCGACGAC |
| C2 | tdTomato_TgTRPPL2_R | tgctcaccatCTCTTCTCCCAGGATGTTG |
| C3 | TgTRPPL2_tdTomato_F | gggagaagagATGGTGAGCAAGGGCGAG |
| C4 | pCDNA3_TgTRPPL2_tdTomato_R | acactatagaatagggccctCTACTTGTACAGCTCGTCC ATG |
| C5 | TgTRPPL2_Validation_F | GCAAGAAGAAGAAACGACGCAAG |
| C6 | TgTRPPL2_Validation_R | CTTTGAGGTCCTAGTTCACCTCCGA |
