# Supplementary material for: Calcium signaling through a transient receptor channel is important for Toxoplasma gondii growth
Source: eLife. 2021 Jun 9;10:e63417. doi: 10.7554/eLife.63417 (PMC8216714; doi:10.7554/eLife.63417)
Supplement: Supplementary file 4. [file elife-63417-supp4.docx]

**Calcium signaling by a Transient Receptor Channel is important for *Toxoplasma gondii* growth**

**Márquez-Nogueras et al**

**Supplementary File 4.** Composition of the solutions used for the electrophysiological analysis.

| **Reagents** | **Concentration (mM)** | | | | |
| --- | --- | --- | --- | --- | --- |
|  | **Solution A** | **Solution B** | **Solution C** | **Solution D** | **Solution E** |
| **KCl** | 140 | 140 | 140 | - | - |
| **CsCl** | - | - | - | 140 | 140 |
| **EGTA** | 0.1 | 0.1 | 0.1 | 0.1 | 0.1 |
| **Intracellular Ca^2+^** | 1.8 | 10 | 1.8 | 1.8 | 10 |
| **Extracellular Ca^2+^** | 0.0001 | 0.0001 | 0.01 | 0.0001 | 0.0001 |
